# Efficacy of Video-Assisted Instruction on Knowledge and Performance of Dental Students in Access Cavity Preparation

**DOI:** 10.22037/iej.2016.14

**Published:** 2016

**Authors:** Mandana Naseri, Yazdan Shantiaee, Javid Rasekhi, Saeede Zadsirjan, Maryam Mojtahed Bidabadi, Akbar Khayat

**Affiliations:** a *Department of Endodontics, Dental School,Shahid Beheshti University of Medical Sciences, Tehran, Iran; *; b * Oral Medicine Department, Dental School, Mashhad University of Medical Sciences, Mashhad, Iran; *; c * Department of Endodontics, Dental School, Hamadan University of Medical Sciences, Hamadan, Iran;*; d * Division of Endodontics, Department of Oral Biological and Medical Sciences, Dental School, University of British Columbia, Canada*

**Keywords:** Conventional Education, Endodontic Treatment, Knowledge, Performance, Video-Assisted

## Abstract

**Introduction:**

The conventional method of teaching endodontics has some drawbacks. Due to the small size of the oral cavity, students cannot closely observe the clinical procedure. Use of new teaching modalities such as the intraoral camera may obviate this problem. This study assessed the effect of video-assisted clinical instruction in dentistry (VACID) on dental student’s knowledge and performance in access cavity preparation during endodontic treatment.

**Methods and Materials:**

In this interventional study, twenty six undergraduate students were equally divided into two groups and received instructions on access cavity preparation *via* conventional demonstration (CD) or VACID using intraoral camera plus conventional demonstration. Students’ knowledge was assessed before and after the demonstration. The scores obtained by students were compared between the two groups. Data were analyzed using the Mann Whitney U test.

**Results:**

No significant difference was found between the two groups in knowledge and performance scores of students about pulp chamber removal, under-extension, over-extension, gouging, perforation or finding the main and extra canals. However, use of intraoral camera significantly reduced the number of student visits to instructors for problem solving (*P*=0.001).

**Conclusion:**

VACID is an effective educational method and as efficient as conventional demonstration in endodontics; as a result it can be used in combination with conventional teaching.

## Introduction

Traditionally, the science of endodontics has been taught to dental students through live demonstration. In this method, the mentor performs the treatment step by step in the presence of students while they watch the entire process. Teaching clinical procedures *via* live demonstrations has shown to be effective in increasing the self-confidence of students, their communication skills and better perception of clinical procedures [1]. However, this technique has some drawbacks. Due to the large number of students and small working environment (oral cavity), not all the students get to see the entire procedure. This method is also very time-consuming. Moreover, although clinically efficient, technical aspects of the therapeutic procedures are often not well discussed during these demonstrations [2]. Thus, medical education programmers have been searching for new teaching techniques to enhance the dental learning process especially in clinical fields like endodontics. 

Observation of treatment process by students is important. Improving this process in any way reduces the risk of procedural errors. Several techniques have been suggested for improving the teaching process such as recording the treatment steps for future reference and on-screen demonstration review of the treatment process using an intraoral camera. However, efficacy of these systems has to be confirmed through clinical trials. 

Video-assisted clinical instruction in dentistry (VACID) is an educational tool that uses video-images to complement dental education. Benefits of this technique in improving technical skills [3] and simulation of clinical setting [4] have been confirmed. Also, students have shown high acceptability towards this technique [4]. The superiority of VACID over traditional teaching for education of clinical skills has been reported in several studies [5] and is probably attributed to the better observation of clinical procedures and improved interpretation of details. Some authors have suggested it as a supplement to conventional demonstration [6] and mentioned its positive role in enhancement of the clinical procedure [7]. Fakhry *et al.* [8] evaluated VACID in the clinical setting at the Department of Periodontics of Iowa and reported that this educational modality improves the real-time visualization of periodontal procedures significantly more than conventional observation.

This study aimed to evaluate the efficacy of educational aids on knowledge and performance of dental students in access cavity preparation during endodontic treatment.

## Materials and Methods

A total of 26 undergraduate students taking elementary clinical endodontic course at Shahid Beheshti Dental School participated in this study. The students were randomly divided into two groups: group one (*n*=13) was taught by conventional demonstration (CD) and the students observed the progress of the clinical procedure directly in the mouth and group two (*n*=13) were taught by conventional lecture and VACID. A pre-test was held to assess the baseline information of students about access cavity preparation by demonstrating the clinical slides to students and scoring their responses to questions. After the pretest, group 1 (CD) received a conventional practical demonstration of access cavity preparation on a patient and students directly observed the steps of treatment. Group 2 (VACID) students received a practical demonstration on a patient but the entire procedure was displayed step-by-step on a monitor close to the dental unit using an intraoral camera and the associated software program easily seen by all students. The provided teaching included complete instruction of access cavity preparation in maxillary and mandibular molars, canal negotiation and searching for the second mesiobuccal canal (MB2) in maxillary molars. Two instructors operated on patients and specific guidelines were given to them in order to match the demonstrations. The criteria for evaluation were pulp chamber roof removal, prevention of under-extension, over-extension, gouging and perforation errors, finding main canals and extra canals. The given scores to students were calibrated as 2, 1 and 0 for good, moderate and poor performance, respectively. Also, after instruction, performance of students during access cavity preparation in the two groups were clinically evaluated and scored by instructors blinded to the group allocation of students. A post-test was held to assess the students’ knowledge level. Number of referring to the instructors by students were compared between the two groups. Non-parametric Mann Whitney U test was applied for the comparison of the two groups. The Wilcoxon signed rank test was used for pairwise comparison of groups before and after interventions. Analysis of pre-test and post-test data was done by ANOVA considering the confounding factor as “between subject comparison” and pretest score as the covariate. 

## Results

Table 1 shows the result of students’ knowledge evaluation *via *pre- and post-test. According to the results of the Mann Whitney U test no significant difference was found in terms of access cavity preparation between the two groups. ANOVA failed to show a significant difference in access cavity preparation in “between the subject” comparison (*P*>0.05).

Table 2 shows the result of students’ performance. The only significant difference was found in number of student referrals to instructors for problem solving which was significantly less in VACID group (*P*=0.001).

## Discussion

Clinical demonstrations in dental education have been shown to be less rewarding as the result of limited visual access to the demonstrated treatment. In this study we compared the efficacy of traditional clinical demonstrations with broadcasted demonstration of access cavity preparation. An intraoral camera provided a magnified view of the procedure carried out by the instructor. In knowledge and practical skills for access cavity preparation, significant differences were found between the two groups. However, the number of referrals to instructors for problem solving was significantly less in the intervention group. This is an important finding because less referral improves the performance of instructors and prevents fatigue. 

**Table 1:** Mean (SD) of pre-test and post-test scores based on type of instruction

**Type of instruction (N)**	**Mean (SD)**	
	**Pre-test**	**Post-test**
**CD (13)**	77 (43)	85 (37)
**VACID (13)**	46 (51)	54 (51)

**Table 2:** Mean (SD) scores of students during access cavity preparation

	**Mean (SD)**							
	**Pulp chamber roof removal**	**Under-extension**	**Over-extension**	**Gouging**	**Perforation**	**Finding main canal**	**Finding extra canals**	**Referral to instructor**
**CD**	1.08 (0.49)	1.54 (0. 66)	1.69 (0.48)	1.77 (0.43)	1.92 (0.27)	1.54 (0.51)	1.92 (0.27)	3.54 (0.87)
**VACID**	1.23 (0.59)	1.69 (0.63)	1.92 (0.27)	1.92 (0.27)	2 (0.00)	1.85 (0.37)	1.92 (0.27)	2.23 (0.59)

Dental education needs modern learning modalities and technologies. In dentistry, VACID is based on using video technology in order to enhance the technical skills and knowledge acquisition of dental students. This teaching modality is student-centered and improves students’ critical self-appraisal. In this technique, video images are captured and displayed live on a monitor or screen. Video images can also be recorded for future referencing. 

Rystedt *et al.* [5] evaluated video-based clinical demonstrations in comparison with conventional demonstration in preclinical dental teaching and reported that video-based demonstrations provided an opportunity for the students to integrate their theoretical and clinical knowledge; which was attributed to the improved visualization and more attention to details because of better observation.

Packer *et al.* [9], evaluated the use of plasma screen technology for dental undergraduate teaching and found that this technology can be a great teaching tool as it provides a much better view of practical procedures. Its close-up views can be a supplement to live demonstration. 

Real time video magnification was evaluated by Robinson and Lee [10], aiming at pre-clinical instruction of crown preparations. Preparations made by students using video magnification were more accurate and the understanding of students was significantly improved by the use of magnification.

Based on the available literature and our study results, VACID seems to be an efficient teaching tool and can be used as an adjunct to conventional demonstration in dental education. It complements the current conventional clinical and didactic teaching curricula.

In another study, Fakhry *et al.* [2] thoroughly discussed VACID and its use in dental education and reported favorable results for this teaching modality in comparison with the traditional teaching system. Application of video-assisted instruction has also been useful in the clinical practice of medicine [11].

Considering our results and the above-mentioned findings regarding the positive effects of VACID and also the fact that this teaching modality is inexpensive, it can be stated that VACID can be easily applied for teaching clinical procedures in dentistry. Future studies with a larger sample size are required to assess VACID on more and variable complicated clinical procedures in dentistry for better elucidation of the positive effects of this teaching modality.

## Conclusion

This study showed that VACID was an effective adjunct to traditional teaching in access cavity preparation as it significantly decreased the number of student referrals to instructors for problem solving. Thus, VACID is recommended for teaching in dental schools.
